# Supplementary material for: Anxiety and depressive symptoms among home isolated patients with COVID-19: A cross-sectional study from Province One, Nepal
Source: PLOS Glob Public Health. 2022 Sep 12;2(9):e0001046. doi: 10.1371/journal.pgph.0001046 (PMC10022042; doi:10.1371/journal.pgph.0001046)
Supplement: S1 Appendix — (DOC) [file pgph.0001046.s001.doc]

S1 Appendix: STROBE Statement—Checklist of items that should be included in reports of ***cross-sectional studies***

|  | Item No. | Recommendation | Page No. | Relevant text from manuscript |
| --- | --- | --- | --- | --- |
| **Title and abstract** | 1 | (*a*) Indicate the study’s design with a commonly used term in the title or the abstract | 2 | Cross-sectional study (Title) |
|  |  | (*b*) Provide in the abstract an informative and balanced summary of what was done and what was found | 2 | The study aimed to identify the prevalence and factors associated with anxiety and depression among COVID-19 home isolated patients in Province One, Nepal. |
| Introduction | | |  |  |
| Background/rationale | 2 | Explain the scientific background and rationale for the investigation being reported | 5 | In our literature review, no previous studies have documented the mental health status of home isolated patients during the COVID-19 pandemic. A better understanding of the psychosocial problems of home isolated patients can provide important guidance in carrying out timely psychological interventions during the COVID-19 pandemic and in any future outbreaks |
| Objectives | 3 | State specific objectives, including any prespecified hypotheses | 5 | This study aimed to determine the prevalence and major influencing factors of anxiety and depressive symptoms among COVID-19 home isolated patients. |
| Methods | | |  |  |
| Study design | 4 | Present key elements of study design early in the paper | 5 | A cross-sectional study was conducted via phone interview among COVID-19 infected patients who had spent at least 5 days at home isolation |
| Setting | 5 | Describe the setting, locations, and relevant dates, including periods of recruitment, exposure, follow-up, and data collection | 5 | The study participants were from Province One, Nepal. Data were collected between February 17, 2021, and April 9, 2021. |
| Participants | 6 | (*a*) Give the eligibility criteria, and the sources and methods of selection of participants | 6 | The sampling frame of COVID-19 home isolated patients was available from the database developed by the Ministry of Social Development of Province One, Nepal. A simple random sampling technique using the RAND command in Microsoft Excel was used to select the participants from the sampling frame of 11543 COVID-infected patients. |
| Variables | 7 | Clearly define all outcomes, exposures, predictors, potential confounders, and effect modifiers. Give diagnostic criteria, if applicable | 6-7 | The anxiety and depression status of the participants were assessed which were the outcome variables. The socio-demographic variables included sex, age, education, family type, occupation, and marital status. COVID-19 and behavioral related characteristics consisted of information of participants having health workers in family members, presence of COVID-19 symptoms, presence of comorbidity, complication during COVID-19, taking medicine for COVID-19 symptoms, use of the internet during isolation, watching TV during isolation, use of social media for COVID-19 information, use of Ministry of Health and Population (MOHP)/WHO site for COVID-19 information, smoking and alcohol history. |
| Data sources/ measurement | 8* | For each variable of interest, give sources of data and details of methods of assessment (measurement). Describe comparability of assessment methods if there is more than one group | 6 | The anxiety and depression status of the participants were assessed using the 14-item Hospital Anxiety and Depression Scale (HADS). All confirmed home isolated patients were contacted by telephone with an invitation to join the study. Those providing consent were interviewed using a structured questionnaire in their convenient time. |
| Bias | 9 | Describe any efforts to address potential sources of bias | 6,8 | Participants were selected randomly from a sampling frame. Enumerators with previous data collection experience and academic background in public health were recruited and trained by the study team. The study objectives were carefully explained to the study objectives before the interview. |
| Study size | 10 | Explain how the study size was arrived at | 6 | The sample size was calculated using the formula of a cross-sectional survey. |
| Quantitative variables | 11 | Explain how quantitative variables were handled in the analyses. If applicable, describe which groupings were chosen and why | 7 | Descriptive analysis was done by calculating frequency and percentages for categorical variables |
| Statistical methods | 12 | (*a*) Describe all statistical methods, including those used to control for confounding | 7 | The Chi-square test was used to determine the association between categorical variables. |
|  |  | (*b*) Describe any methods used to examine subgroups and interactions | 7 | To determine potential factors associated with the outcome variable, a multivariable logistic regression analysis was performed, and adjusted odds ratio (AOR) and 95% confidence interval (CI) were calculated. |
|  |  | (*c*) Explain how missing data were addressed |  | NA |
|  |  | (*d*) If applicable, describe analytical methods taking account of sampling strategy |  | NA |
|  |  | (*e*) Describe any sensitivity analyses |  | NA |
| Results | | |  |  |
| Participants | 13* | (a) Report numbers of individuals at each stage of study—eg numbers potentially eligible, examined for eligibility, confirmed eligible, included in the study, completing follow-up, and analysed | 8 | A total of 372 participants were recruited in the study |
|  |  | (b) Give reasons for non-participation at each stage |  | NA |
|  |  | (c) Consider use of a flow diagram |  | NA |
| Descriptive data | 14* | (a) Give characteristics of study participants (eg demographic, clinical, social) and information on exposures and potential confounders | 9-12 | See Table 2 and Table 3 |
|  |  | (b) Indicate number of participants with missing data for each variable of interest |  | NA |
| Outcome data | 15* | Report numbers of outcome events or summary measures | 8-9 | Out of 372 participants, 74.2% (n=276) had symptoms of anxiety (borderline: 48.7% and abnormal: 25.5%). Similarly, 79% (n=294) of the participants experienced symptoms of depression (borderline: 52.7% and abnormal: 26.3%). |
| Main results | 16 | (*a*) Give unadjusted estimates and, if applicable, confounder-adjusted estimates and their precision (eg, 95% confidence interval). Make clear which confounders were adjusted for and why they were included | 13-14 | See Table 4 |
|  |  | (*b*) Report category boundaries when continuous variables were categorized | 7 | For further analysis, a score of more than 7 was considered as the presence of anxiety and depression |
|  |  | (*c*) If relevant, consider translating estimates of relative risk into absolute risk for a meaningful time period |  | NA |
| Other analyses | 17 | Report other analyses done—eg analyses of subgroups and interactions, and sensitivity analyses | 13-14 | Multivariable regression model was used. See Table 4 |
| Discussion | | |  |  |
| Key results | 18 | Summarise key results with reference to study objectives | 14-15 | The prevalence of anxiety and depressive symptoms was found in the majority of the home isolated patients with more than half having borderline symptoms and one in four having abnormal symptoms. |
| Limitations | 19 | Discuss limitations of the study, taking into account sources of potential bias or imprecision. Discuss both direction and magnitude of any potential bias | 17 | This study was conducted during the early phase of the pandemic when treatment and vaccines were not available and thus could have affected the presence of anxiety and depression symptoms. Similarly, mental health outcomes might still reflect conditions existing before this pandemic. Besides, the present study lacked clinical interviews to confirm the diagnosis of anxiety and depression. Also, we have not included the history of mental illness and medications taken for any kind of mental illness before the pandemic. |
| Interpretation | 20 | Give a cautious overall interpretation of results considering objectives, limitations, multiplicity of analyses, results from similar studies, and other relevant evidence | 18 | In summary, the findings have shown that a substantial proportion of COVID-19 patients in Nepal experienced depressive and anxiety symptoms during home isolation with more than half having borderline and one out of four having abnormal mental health symptoms. Female and those with COVID-19 symptoms had higher odds of exhibiting depression symptoms while ever married, those with COVID-19 related complications and those who took medicines for treatment of symptoms were at higher odds of developing anxiety symptoms. Interestingly, watching Television during isolation was associated with lower odds of developing anxiety and depression symptoms |
| Generalisability | 21 | Discuss the generalisability (external validity) of the study results | 17 | The study being based in Province One may not be generalizable to study population from other provinces. |
| Other information | | |  |  |
| Funding | 22 | Give the source of funding and the role of the funders for the present study and, if applicable, for the original study on which the present article is based | 18 | The study did not receive any funding for this work. |

*Give information separately for exposed and unexposed groups.

**Note:** An Explanation and Elaboration article discusses each checklist item and gives methodological background and published examples of transparent reporting. The STROBE checklist is best used in conjunction with this article (freely available on the Web sites of PLoS Medicine at http://www.plosmedicine.org/, Annals of Internal Medicine at http://www.annals.org/, and Epidemiology at http://www.epidem.com/). Information on the STROBE Initiative is available at www.strobe-statement.org.
