# Supplementary material for: Anxiety and depressive symptoms among home isolated patients with COVID-19: A cross-sectional study from Province One, Nepal
Source: PLOS Glob Public Health. 2022 Sep 12;2(9):e0001046. doi: 10.1371/journal.pgph.0001046 (PMC10022042; doi:10.1371/journal.pgph.0001046)
Supplement: Attachment — Submitted filename: Response to Reviewers.docx [file pgph.0001046.s002.docx]

**July 31, 2022**

To,

Editorial Board

Plos Global Public Health

**Subject: Submission of revised manuscript titled ‘Anxiety and depressive symptoms among home isolated patients with COVID-19 in a low-resource setting: a cross-sectional study from Nepal’**

The study team would like to thank reviewers for providing their feedback on the manuscript and we are confident that the reviewer’s comment has helped to improve the quality of the manuscript. Please find the response to the reviewer’s comment below.

Reviewer #1:

**Congratulations on completing your work and disseminating the results. This manuscript relevant to the current situation in the midst of the Covid-19 pandemic. I have some feedback intended to be helpful, and I wish you the best of luck in your ongoing research. Take care**

Thank you very much for your feedback.

**Introduction
- I found that the subject matter was important, and the knowledge gap is presented in the introduction.**
**- A small but relatively significant comparison revealed that the purpose as outlined in the abstract and the body of the paper are different (please make this consistent).**

Thank you for flagging this issue, we have now have addressed this issue in the manuscript.

**Method
- Regarding “COVID-19 patients who had spent at least 5 days at home isolation”, please put the rationale for this approach.**

During the time of the survey, patients with COVID-19 need to stay at home isolation for 14 days. The study team assumed that it would be logical to include those participants who had spent at least 5 days at home isolation in the study considering that they would be exposed to the isolation environment.

**- Please kindly put the information about psychometric properties such as validity and reliability all of the instruments.**

Thank you for the suggestion. We have included a statement about the psychometric properties of the Nepali version of the tool.

**Discussion
- If possible, please add an alternative explanation regarding study finding about female participant had higher odds from cultural perspective in your country.**

Thank you for the suggestion. We have added a statement which could support the argument that females in Nepal have higher odds of developing depressive symptoms.

**Conclusion
-Some of your conclusion is not coherent with your findings, please make sure answer the research question in your conclusion.**

We now have made our conclusion coherent with our findings and we have answered the research question.

**Reviewer #2: This is an important piece of work, demonstrating the mental health burden among individuals living in home isolation after testing positive for COVID-19 in Nepal. Insofar as I wish that researchers could have expanded the geography to include other parts of the country and compared the mental health burden between home-isolated versus the general population, I understand the challenges in obtaining primary data for the study with a broader scope. Still, I find this research useful, and within the scope of this journal.**

Thank you for appreciating our task. Studies so far have focused on general population, institution-based isolated patients, and specific population such as health workers. However, mental health status among home-isolated COVID-19 patients have not been explored and thus this study focused on home-isolated patients and not on general population. This study covers participants from one out of seven provinces of Nepal which is the limitation of this study.

**The study is generally well-presented, is relatively straightforward, and has used an easy but established methodology. Below are some of my comments, many of them being minor. I would be happy to take a revised version of this manuscript.**

**Title: I can understand the authors’ temptation to highlight “low resource settings” for this story conducted in Nepal but I note that this study is neither nationally representative nor conducted in the most impoverished geography within Nepal (In fact, province one is among the one with better HDI). Moreover, the poor-resource setting is not much discussed in the manuscript text. Therefore, I would recommend dropping the word “low resource settings” from the title.**

Thank you for your suggestion. We have removed low resource settings from our study title and have specified the name of the province.

**Abstract: I think it makes sense to move up the sentence starting in line 42. This way, the abstract reads more coherent: first, you highlight poor mental health is a big issue and then you study factors contributing to it. This is also the order results are presented in the main text.**

Thank you for the suggestion. We initially have placed this sentence as a concluding sentence. We now have removed it as the burden of anxiety and depressive symptoms have already been covered in the starting of the results.

**In line 34, I would recommend being more specific and replacing “multivariable analysis” with “multivariable logistic regression analysis”.**

Done

**Also, in line 34, to the casual readers, it’s confusing what 0-21 means. So, just remove “(HADS:0-21)”.**

Done

**In Line 44: It is not clear what “connection to the health system” means. I think that the significance of this result holds whether or not the home isolations are tied to government-imposed forced quarantines or self-quarantine. If authors want to restrict their inference to a particular type of quarantine scheme, please make this sentence a bit clearer.**

Thank you for the suggestion. We have replaced ‘connection to the health system’ with ‘connection to health services’ as home-isolated patients are not linked with health facilities in case of emergency or for seeking information.

**Keywords: Mental Health could be another potential keyword here.**

Done.

**Introduction: This section can be tightened in a few places. For example, for this study, it is more important to highlight when the Covid case/death was first reported in Nepal and Province 1, rather than pretty-much-known info about Wuhan.**

Thank you for the suggestion. We have included information about first reported COVID-19 infection and death in Nepal. We have removed much known information about Wuhan.

**On line 62: Please re-phrase the sentence “The onslaught of ….” so that this statement looks relevant years from now.**

We have decided to remove the sentence since this might be irrelevant considering the study population.

**Line 71: This sentence “A study from Saudi Arabia reported 17.1%...” reads awkwardly. Rephrase this one, emphasizing that these numbers are prevalence estimates.**

Done

**On line 78: white space in “ofNepal”**

Done

**On line 82: I don’t think “close supervision of local health workers” is accurate. It was one of the haphazard, ad-doc decisions mostly carried out by the local government. I know you cited a gov report but they should be taken with a grain of salt.**

Thank you for your concern. We placed this statement as per the home isolation guideline. During the period of the survey, health workers used to contact home isolated patients for monitoring their health status.

**Line 86: I would not use “could have been” because it usually means something was possible in the past but did not happen.**

We used this word because we did not know about the burden of the situation and this was a study hypothesis. As the study is cross-sectional, the findings capture only the moment when the study was carried out.

**Line 87: change “During” to something like “As of”. I get what you trying to say here but reads a little off.**

We used ‘In our literature review’ instead of during.

Methods:
**Line 95: I am not a clinician, but I’d probably not use the term “patients” here and elsewhere since these are the individuals who tested positive for the virus (and not necessarily showing symptoms of the disease).**

Thank you. COVID-19 infected patient is a common word in literature, and hence we used this word.

**Line 96: It might be helpful for international readers to add a little context to the province you are studying**

Thank you very much for the suggestion. We now have added a little context to the study province.

**Line 99: white space in “2021and”**

Done

**Line 104: Missing hyphen in “RT PCR”**

Done

**In the sample size calculation, a general comment: it is important to note that you are estimating the minimum sample size required to draw a reasonable inference. It is always preferable to use more information when possible. In other words, the number you obtained by plugging your assumptions (i.e. 372) is not a magic number that can’t be exceeded in selecting respondents, and when possible, aim to recruit more participants.**

We agree to your opinion regarding the sample size. However, in our study, we recruited the minimum sample size required for the study.

**Line 111: white space in “of372”**

Done

**Line 121: “which are scored from 0 to 21”: Either mention that it’s the “total” score that ranges from 0 to 21, or write that each individual question is scored from 0 to 3.**

Done

**Line 125: Please try to rephrase “study tools” in this context.**

Thank you. We rephrased ‘study tools’ with questionnaire.

**Line 127: comma not needed in (Nuclear, and Joint/Extended)**

Done

**Line 137: Chi-square test does not distinguish “independent” or “dependent” variables. Just say categorical variables.**

Done

**Double check whether you adjusted for chronic comorbidities for both anxiety and depression. In the methods, you say this goes into models for both outcomes but the result table says otherwise.**

Thank you for pointing out. We adjusted presence of chronic comorbidities only for anxiety since this variable was significant at 10% level of significance. We have rectified this in method.

**Results: The exceptionally high prevalence, particularly for depression, made me double-think about the validity of the instrument used to identify these outcomes and the willingness/understanding/wording during the interview process. A vast majority have a borderline problem, perhaps indicating central tendency bias of respondents. Assuming that people with borderline health problems could be cared for with little resources than those with chronic/serious MH patients, it suggests that even the smaller efforts put into mental health could go a long way. I think that this could be something authors can consider discussing and calling for action in their discussion/conclusion.**

Thank you for the suggestion regarding the call for action for dealing with borderline mental health symptoms. We have now incorporated this in the conclusion section.

**Table 4. One way to reduce the unnecessary rows is to specify the reference category when you mention the variable name. It makes the table easier to read and avoids countless Ref/Ref.**

Thank you for the feedback. We now have revised the variable by specifying the reference category next to the variable name.

**I see no value in displaying crude ORs. These can be easily derived from the characteristics of the respondent’s tables displayed previously. But it’s up to the authors whether to keep it or not.**

Thank you for the suggestion. We now have removed information regarding crude ORs in variables which was not adjusted in the regression model.

**Discussion: The authors do a fairly good job in this section, but I wanted to see a more nuanced and focused discussion around the mental health context in Nepal and what authorities and researchers should do next. For example, please put the research in the broader context of mental health problems—Nepal is one of the few countries that saw an increase in the suicide rate during the pandemic. (**[**https://journals.plos.org/plosone/article?id=10.1371/journal.pone.0262958**](https://journals.plos.org/plosone/article?id=10.1371/journal.pone.0262958)**). This research can be easily molded to the existing body of knowledge from within Nepal (I don’t see that paper in the reference).**

Thank you for the suggestion. We have added more context in the discussion around the mental health context in Nepal, and also cited the article.

**Given the worrying mental health prevalence in province 1, how important is it to take this issue seriously in other provinces that are even poorer? What proportions of respondents were in imposed quarantine facilities or how many were in their own homes? Would the elevated mental health problems among women might indicate the forced quarantines were not women-friendly? Maybe authors might comment something on TV use and better mental health?**

As the study was limited to Province One, we can only assume that the situation could be worse in other provinces with low socio-economic status. All the respondents were at home isolation and were not necessarily forced quarantines. We already have dealt about TV use and its relationship with mental health outcomes in the discussion section.

Thank you

Pratik Khanal (on behalf of the study team)

Corresponding author

[pratikkhanal@iom.edu.np](mailto:pratikkhanal@iom.edu.np)
