# Supplementary material for: Anxiety and depressive symptoms among home isolated patients with COVID-19: A cross-sectional study from Province One, Nepal
Source: PLOS Glob Public Health. 2022 Sep 12;2(9):e0001046. doi: 10.1371/journal.pgph.0001046 (PMC10022042; doi:10.1371/journal.pgph.0001046)
Supplement: Attachment — Submitted filename: Response to Reviewers.docx [file pgph.0001046.s003.docx]

**August 13, 2022**

To,

Editorial Board

Plos Global Public Health

**Subject: Submission of revised manuscript (PGPH-D-22-00768R1) titled ‘Anxiety and depressive symptoms among home isolated patients with COVID-19: a cross-sectional study from Province One, Nepal’**

The study team would like to thank editor and reviewers for providing their feedback on the manuscript and we are confident that the reviewer’s comment has helped to improve the quality of the manuscript. Please find the response to the editorial and reviewer’s comment below.

Journal Requirements:

**Please review your reference list to ensure that it is complete and correct. If you have cited papers that have been retracted, please include the rationale for doing so in the manuscript text, or remove these references and replace them with relevant current references. Any changes to the reference list should be mentioned in the rebuttal letter that accompanies your revised manuscript. If you need to cite a retracted article, indicate the article’s retracted status in the References list and also include a citation and full reference for the retraction notice.**

Response: We have reviewed the reference list and ensured that there are no retracted papers. We have replaced a pre-print paper with the published paper in the discussion section.

Gamonal-Limcaoco S, Montero-Mateos E, Lozano-López MT, Maciá-Casas A, Matías-Fernández J, Roncero C. Perceived stress in different countries at the beginning of the coronavirus pandemic. Int J Psychiatry Med [Internet]. 2021 Jul 16;57(4):309–22. Available from: <https://doi.org/10.1177/00912174211033710>

Similarly, we have included a relevant reference in the introduction section of the manuscript.

BC UB, Pokharel S, Munikar S, Wagle CN, Adhikary P, Shahi BB, Thapa C, Bhandari RP, Adhikari B, Thapa K. Anxiety and depression among people living in quarantine centers during COVID-19 pandemic: A mixed method study from western Nepal. PloS one. 2021 Jul 9;16(7):e0254126.

**Please add a supplementary file indicating your study adheres to STROBE guideline for cross-sectional studies. You can access a copy of the guideline can be accessed via this link** [**Best Practices in Research Reporting | PLOS Global Public Health**](https://journals.plos.org/globalpublichealth/s/best-practices-in-research-reporting)
Response: Thank you very much. We now have added a supplementary file indicating that our study adheres to STROBE guideline for cross-sectional studies. Please check the Supplementary File.

**Reviewer #1: The authors have done an excellent job responding to reviewer feedback, both mine and that of the other reviewers. I was able identify my comments and how they were addressed, as well as that of the others. I have no follow-up recommendations and suggest accept without further revision. Congratulations on an excellent response.**

**Reviewer #2: Thank you for addressing all the concerns and thorough revision. I look forward for the positive editorial decision in this important piece of work.**

Response: Thank you both Reviewer 1 and Reviewer 2 for your suggestions which was insightful and helpful to improve our work.

Due to duplicate account in the Plos One, I am not able to authenticate my ORCID ID. I would like to request the journal to include my ORCID ID as <https://orcid.org/0000-0002-1057-5700>

On behalf of the study team, I would like to thank you for your feedback, and we hope that we have addressed all of your suggestions in the revised manuscript.

Thanking you

Pratik Khanal

Corresponding author

[pratikkhanal@iom.edu.np](mailto:pratikkhanal@iom.edu.np)
